# Concordant Patterns of Population Genetic Structure in Food-Deceptive *Dactylorhiza* Orchids

**DOI:** 10.3390/genes16010067

**Published:** 2025-01-08

**Authors:** Ada Wróblewska, Beata Ostrowiecka, Edyta Jermakowicz, Izabela Tałałaj

**Affiliations:** Faculty of Biology, University of Bialystok, Ciołkowskiego 1J Street, 15-245 Białystok, Poland; b.ostrowiecka@uwb.edu.pl (B.O.); edytabot@uwb.edu.pl (E.J.); izagry@uwb.edu.pl (I.T.)

**Keywords:** *Dactylorhiza fuchsii*, *Dactylorhiza incarnata* var. *incarnata*, *Dactylorhiza majalis*, *F*_IS_, spatial genetic structure

## Abstract

Background: The patterns of inbreeding coefficients (*F*_IS_) and fine spatial genetic structure (FSGS) were evaluated regarding the mating system and inbreeding depression of food-deceptive orchids, *Dactylorhiza majalis*, *Dactylorhiza incarnata* var. *incarnata*, and *Dactylorhiza fuchsii*, from NE Poland. Methods: We used 455 individuals, representing nine populations of three taxa and AFLPs, to estimate percent polymorphic loci and Nei’s gene diversity, which are calculated using the Bayesian method; *F*_IS_; *F*_ST_; FSGS with the pairwise kinship coefficient (*Fij*); and AMOVA in populations. Results: We detected a relatively high proportion of polymorphic fragments (40.4–68.4%) and Nei’s gene diversity indices (0.140–0.234). The overall *F*_IS_ was relatively low to moderate (0.071–0.312). The average *F*ij for the populations of three *Dactylorhiza* showed significantly positive values, which were observed between plants at distances of 1–10 m (20 m). *F*_ST_ was significant in each *Dactylorhiza* taxon, ranging from the lowest values in *D. fuchsii* and *D. majalis* (0.080–0.086, *p* < 0.05) to a higher value (0.163, *p* < 0.05) in *D. incarnata* var. *incarnata*. Molecular variance was the highest within populations (76.5–86.6%; *p* < 0.001). Conclusions: We observed concordant genetic diversity patterns in three food-deceptive, allogamous, pollinator-dependent, and self-compatible *Dactylorhiza*. *F*_IS_ is often substantially higher than *F*ij with respect to the first class of FSGSs, suggesting that selfing (meaning of geitonogamy) is at least responsible for homozygosity. A strong FSGS may have evolutionary consequences in *Dactylorhiza*, and combined with low inbreeding depression, it may impact the establishment of inbred lines of *D. majalis* and *D. incarnata* var. *incarnata*.

## 1. Introduction

The mating system influences the genetic structure of plant populations by altering the drift/migration equilibrium, which is defined by the effective population size [1,2]. Theoretical and experimental studies have frequently shown that pollen transfer in outcrossing species results in lower genetic structure and higher genetic diversity than in self-pollinating species. In the latter, it is often attributed to a significant founder effect, which tends to increase inbreeding in selfers [2,3,4]. However, pollen flow may act in concert with life-history traits, such as dispersal mechanisms (by wind or animals), and combined, they exhibit high statistical power for predicting the scale of the fine spatial genetic structure (FSGS) within populations [5,6]. When pollen and seed dispersal are restricted, resulting in significant intra-population structure, biparental inbreeding can also influence the inbreeding coefficient. Therefore, following an isolation-by-distance model, a strong FSGS is frequent even within allogamous or potentially allogamous plants [6]. Higher levels of FSGS have also been highlighted for selfing and clonal species in low-density populations [6,7,8]. Variations in mating systems and different soil and climatic conditions may additionally contribute to different FSGS patterns [9]. *F*_IS_ also reflects inbreeding in previous generations of perennials, resulting in the Wahlund effect on the population [10]. Mating systems and seed dispersal also influence *F*_ST_ via its impact on pollen-mediated and short and/or leptokurtic gene flow and the effective population size, especially during selfing and mating between relatives, by increasing inbreeding, which enhances genetic drift. A summary report by Duminil et al. [11], which analyzed data from 263 plant species, indicated that the inbreeding coefficient (*F*_IS_) observed at the adult plant stage enables the assessment of the effects of both biparental inbreeding and inbreeding depression on population genetic structure. In the early stages of the plant life cycle, inbreeding depression primarily impacts inbred progeny. As a result, the *F*_IS_ of adult plants reflects information about both the selfing rate and inbreeding depression.

The mating system and gene flow within and among populations in Orchidaceae can generate common genetic diversity patterns and FSGSs [12]. Pollinator-mediated gene flow among populations, e.g., was higher in deceptive than in rewarding orchids [12,13]. The deceived pollinators generally visit only a limited number of flowers among plants within populations, facilitating cross-pollination and decreasing the chances of inbreeding [14,15,16,17,18,19]. Therefore, it can be hypothesized that the FSGS of these orchids is weak. In contrast, within-population genetic structure could be stronger in rewarding ones due to geitonogamy and mating among close relatives. However, based on earlier experimental orchid studies, dusty-like seed dispersal was usually limited, e.g., refs. [20,21,22,23,24,25]. These results agree with studies that have investigated the FSGSs of both deceptive orchids—e.g., *Caladenia tentaculata* [26], *Cephalanthera longibracteata* [22], *Orchis cyclochila* [23], *Orchis purpurea* [27], *Orchis mascula* [28,29], and *Cymbidium goeringii* [30]—and rewarding ones—e.g., *Gymnadenia conopsea* [31], *Pogonia ophioglossoides* [32], and *Epipactis thunbergii* [33]. Moreover, orchid germination success has been reported to be higher in the vicinity of mother plants because a mycorrhiza could favour the establishment of seedlings [34,35].

In this study, we focused on *Dactylorhiza* taxa, which are food-deceptive orchids that do not provide any rewards for their pollinators [36]. This genus can be considered a model due to its plant–pollinator interactions, natural selection, and consequent female reproductive success and its impact on genetic structure in food-deceptive plant groups [37,38,39]. In this context, the mating system and ID in food-deceptive *D. majalis*, *D. incarnata* var. *incarnata*, and *D. fuchsii* populations from NE Poland were documented in detail [40,41,42,43]. A mixed mating system was observed in all three studied *Dactylorhiza* taxa, similarly to Hedrén and Nordström’s study [44]. Ostrowiecka et al. [40] reported that pollinator behaviour in *D. majalis* likely encourages geitonogamy, which accounts for the formation of selfed seeds in fruits at various inflorescence levels, exhibiting germination potential comparable to that of outcrossed seeds within populations. Vallius et al. [45] and Hedrén and Nordstöm [44] proved that different *D. incarnata* varieties were characterized by a high level of inbreeding, and populations might consist of several inbred lines that were fixed for characters, especially with respect to flower colour. Wróblewska et al.’s [42] results corroborate with previous studies on *Dactylorhiza* concerning the low or medium level of fruit sets ranging from 7.4% to 77.5% [36,45,46]. In vitro experiments revealed that the seed germination of three *Dactylorhiza* taxa from both natural pollination and hand treatments (selfing and outcrossing) occurred at a relatively low level, up to 35% (with the exception of *D. fuchsii* and outcrossing experiments) [42]. In vitro asymbiotic seed germination was similar or slightly higher in selfing than crossing experiments in *D. incarnata* var. *incarnata* and *D. majalis*, while it was reversed in *D. fuchsii* [42]. Spontaneous autogamy in three *Dactylorhiza* taxa existed in <1% of pollination in the studied populations and most likely did not affect reproductive success [47,48]. The taxa are characterized as terrestrial, long-lived, self-compatible, tuberous perennial orchids that reproduce primarily via seeds, with vegetative reproduction occurring rarely [40,49]. Pollination is carried out by various insect groups, including Hymenoptera, Diptera, and Coleoptera and predominantly bees and bumblebees [40,41]. Molecular markers such as cpDNA (trnL, trnF, and psbC–trnK), internal transcribed spacer (ITS) sequences, and flow cytometry data have confirmed the taxonomic status of the three orchids studied [41].

Based on the estimates of an earlier ecological survey, e.g., natural fruit sets, a mixed mating system, and inbreeding depression from a controlled crosses treatment, orchid taxa from NE Poland were studied [40,42,43], in addition to the genetic reports of Hedrén and Nordstöm [44] and Naczk et al. [49]. We tested the following hypothesis: inbreeding coefficients are shaped at a high level in food-deceptive orchids *D. majalis*, *D. incarnata* var. *incarnata*, and *D. fuchsii*. We also assumed that seed dispersal mainly occurs over a short distance in orchids that are close to the mother plant, as was observed by many authors who experimentally researched seed dispersal; therefore, fine-scale genetic structure is stronger due to the effect of inbreeding and short-distance dispersal. Finally, the purpose of this study is to (1) estimate the inbreeding coefficient and the intensity of the FSGS using AFLP markers and (2) discuss how similar mating systems and different inbreeding depression shape the genetic diversity patterns of three food-deceptive *Dactylorhiza* taxa.

## 2. Materials and Methods

### 2.1. Study Sites

**Figure 1:**
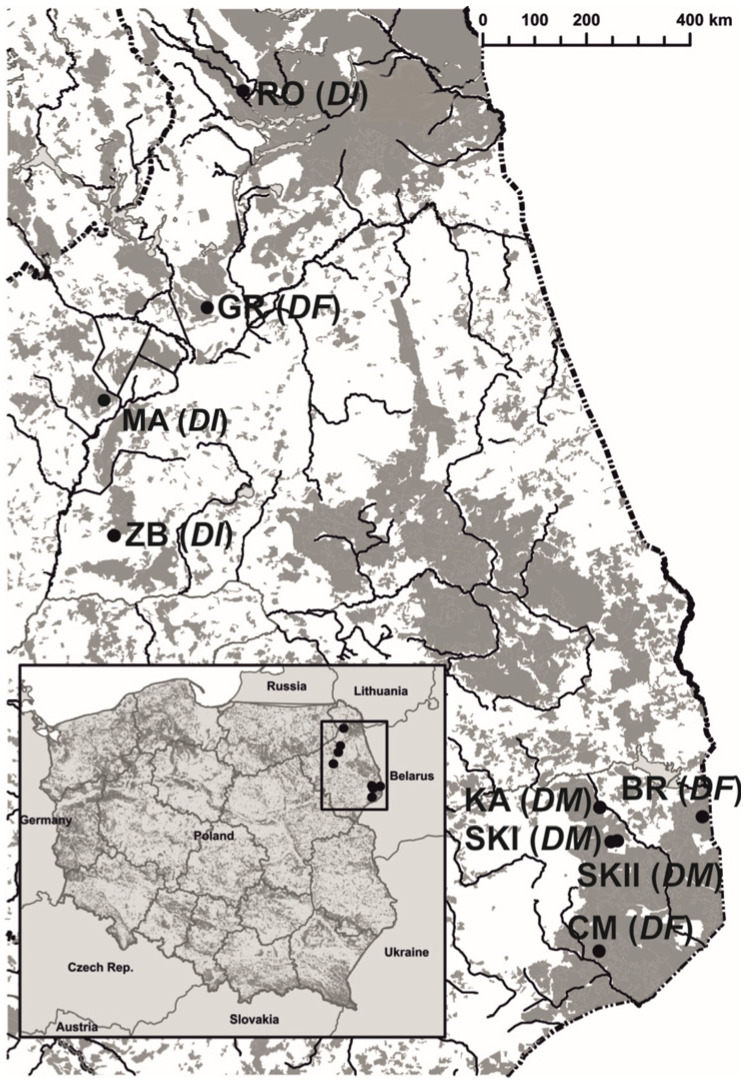
Localities of nine *Dactylorhiza* populations in northeastern Poland. *D. majalis* (*DM*), KA, SKI, and SKII; *D. incarnata* var. *incarnata* (*DI*), ZB, MR, and RO; *D. fuchsii* (*DF*) CM, BR, and GR (Wróblewska et al. 2024a [24]).

The present study was conducted from May to July between 2014 and 2017 across three populations of *D. majalis* (KA, SKI, and SKII), three populations of *D. incarnata* var. *incarnata* (ZB, RO, and MR), and three populations of *D. fuchsii* (BR, CM, and GR) in northeastern Poland (Figure 1). *D. majalis* grows in wet meadows filled with abundant, entomophilous, and rewarding plants. The study sites varied in the number of *D. majalis* individuals, with approximately 120–200 flowering individuals in SKI and SKII and ca. 1000 in KA. All meadows were managed extensively and mowed annually in late July or early August, and they were not subjected to artificial fertilization. The three populations of *D. incarnata* var. *incarnata* were of similar size, with MA having ca. 68–100 flowering plants, ZB with approximately 30–100, and RO with 35–200 (Figure 1). These populations were located in the Biebrza Valley and Rospuda Valley, occupying sedge communities with a low cover of rewarding plant species (ca. 10%). *Dactylorhiza fuchsii* was found in open hornbeam forests with a limited number of rewarding plants, specifically in the Białowieża Primeval Forest and nearby areas (CM and BR, with population sizes of approximately 84–133 flowering plants). One *D. fuchsii* population (GR, with ca. 140–193 flowering plants) was situated in the Biebrza Valley [42].

The study involved samples from 455 individuals across nine populations of three Dactylorhiza taxa, including 162 individuals of *D. majalis* (DM), 129 of *D. incarnata* var. *incarnata* (DI), and 164 of *D. fuchsii* (DF) (Table 1; Figure 1). Although *Dactylorhiza* rarely regenerates clonally, one leaf sample was collected from individual shoots at least 1 m apart within *D. majalis* populations to minimize the effects of population substructure. For *D. incarnata* var. *incarnata* and *D. fuchsii*, samples were collected based on the positions of individuals within their respective populations, which were characterized by different flowering individual densities. Each sample from all populations was mapped using a grid coordinate system with a handheld GPS (Garmin GPSMAP 65s) to facilitate distance calculations between samples.

### 2.2. AFLP Analysis

Genomic DNA was extracted from dried leaf tissues using the Genomic Mini AX Plant kit (A & A Biotechnology, Gdansk, Poland), and the samples were genotyped for AFLP markers. The AFLP procedure, as outlined by Vos et al. [50], was adapted following the Applied Biosystems protocol (AFLPTM Plant Mapping). Initially, 12 primer pair combinations were tested on four selected samples from each *Dactylorhiza* taxon. The GeneScan 500 Liz-labelled size standard (Applied Biosystems, Waltham, MA, USA) was employed for DNA analysis on an ABI 3130. Subsequently, seven primer combinations were selected that yielded polymorphic, clear, and reproducible fragments of consistent intensities across the three *Dactylorhiza* taxa (*D. majalis Eco*R1-ACC/*Mse*I-CAG, *Eco*R1-AGG/*Ms*eI-CAC; *D. incarnata* var. *incarnata Eco*R1-ACA/*MseI*-CAG, *Eco*R1-ACA/*MseI*-CTA; *D. fuchsii Eco*R1-AGG/*Mse*I-CAG, *Eco*R1-ACC/*Mse*I-CAT, *Eco*R1-ACC/*Mse*I-CTA). Variable fragments in the 70–500 bp size range were recorded as present (1) or absent (0) using GeneMapper 4.0 (Applied Biosystems). To assess the repeatability of the AFLP results, three individuals from each population were fully replicated, starting from the restriction/ligation step of the AFLP process. The potential resampling of clones was evaluated using the AFLPdat R-script but was determined to be insignificant and therefore not corrected for.

To evaluate genetic diversity, the proportion of polymorphic fragments (*PL*%) and Nei’s gene diversity (*H*) were calculated using the Bayesian method with a nonuniform prior distribution of allele frequencies as proposed by Zhivotovsky [51] and implemented in AFLP-Surv version 1.0 [52]. The *F* statistic was determined through analysis of molecular variance (AMOVA) using Arlequin 3.11 [53], with the significance of variance components assessed using 1000 independent permutation runs.

The fine-scale genetic structure (FSGS) was analyzed through spatial autocorrelation using the pairwise kinship coefficient *F_ij_* for dominant markers [54]. Mean *F_ij_* estimates for pairs of individuals across specified distance classes were calculated and plotted against distance on a logarithmic scale with SPAGeDi 1.4 [6,54]. Distinct distance classes were created for each population of *D. majalis*, *D. incarnata* var. *incarnata*, and *D. fuchsii* due to varying spatial distribution/density patterns. To evaluate the significance of the FSGS, the regression slopes (*b*) of kinship coefficients against the natural logarithm of distance were compared to slopes obtained from the permutations of individual genotypes (10,000 random permutations). The extent of the FSGS was quantified using the *S*_p_ statistic as proposed by Vekemans and Hardy [6] and calculated as *S*_p_ = −*b*/(1 − *F*_1_), where *b* is the regression slope and *F*_1_ represents the average *F_ij_* between individuals. For each spatial distance class, the 99% confidence interval was determined using 10,000 permutations (with SPAGeDi) [55]. The probability value (*p*) was computed for each spatial distance class and coefficient.

To investigate the *F*_IS_, the Metropolis–Gibbs algorithm was applied in the I4A software based on dominant markers [56]. The data were run using prior values of beat distribution equal to α = β = 1.0 (corresponding to an “uninformative” flat distribution) and 60,000 repetitions, including a 10,000-step burn-in.

## 3. Results

Overall, 193, 215, and 263 polymorphic bands were scored in *D. majalis*, *D. incarnata* var. *incarnata*, and *D. fuchsii*, respectively. Considering the error rates (2%, 1.3%, and 1.5%, respectively), none of the samples may have represented clones.

Relatively high proportions of polymorphic fragments (*PL*% = 40.4–68.4%) and Nei’s gene diversity indices (*H* = 0.140–0.234) were detected among the three orchid species (Table 1). The overall *F*_IS_ was relatively low to moderate, and it equaled 0.071–0.224 in *D. incarnata* var. *incarnata* and 0.079–0.134 in *D. fuchsii*; it reached the highest values of 0.192–0.312 in *D. majalis*.

**Figure 2:**
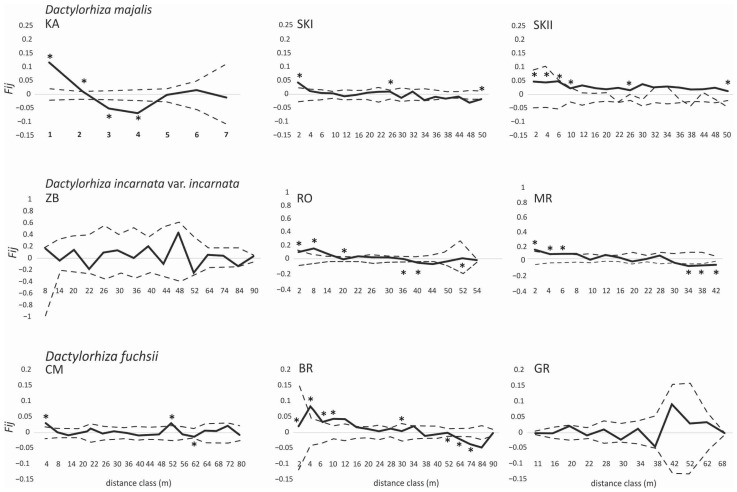
Spatial correlograms for *D. majalis, D. incarnata* var. *incarnata*, and *D. fuchsii* populations with the mean pairwise kinship coefficients (*F_ij_*) of distance classes for AFLPs with respect to the hypothesis of random genetic structure obtained by permuting individual spatial locations, as implemented in SPAGeDi 1.4 [6]. The dotted lines indicate the 99% confidence intervals obtained from 10,000 permutations of genotypes. Codes of populations (KA, SKI, SKII, ZA, MR, RO, CM, BR, and GR; see Table 1); * *p* < 0.05.

The correlograms of the average *F*_ij_ values for the populations of three *Dactylorhiza* taxa exhibited significantly positive values, which were observed in the short-distance classes. In *D. majalis*, the relatives were noted at a distance from 1 m to 10 m (Figure 2), and the values were significantly negative with respect to the longer distance classes (52–72 m) in the two other populations. Similar observations were made in two of the three populations of *D. incarnata* var. *incarnata* and *D. fuchsii.* Significant positive values were observed at a distance from 2 m to 20 m in *D. incarnata* var. *incarnata* (Figure 2) and from 2 m to 10 m in *D. fuchsii* (Figure 2). The *b*_F_ values for *D. majalis* (−0.051–0.009), *D. incarnata* var. *incarnata* (−0.055–0.002), and *D. fuchsii* (−0.026–0.002) were almost all significant (permutation test, *p* < 0.05) (Table 1). The highest *Sp* values were observed for *D. incarnata* var. *incarnata* (0.063) and *D. majalis* (0.056) (Table 1).

Almost all *F*_ST_ values were significant in each *Dactylorhiza* taxon, ranging from the lowest values in *D. fuchsii* and *D. majalis* (0.080 and 0.086, *p* < 0.05, permutation test) to the highest value (0.163, *p* < 0.05, permutation test) in *D. incarnata* var. *incarnata*. The amount of molecular variance was highest within populations, and it was maintained at relatively higher and similar levels in *D. majalis*, *D. incarnata* var. *incarnata*, and *D. fuchsii* (AMOVA: 76.5%, 85.5%, and 86.6%; *p* < 0.001, respectively).

## 4. Discussion

The relationships among mating systems, inbreeding depression, biparental inbreeding, and their effects on *F*_IS_ and *F*_ST_ have been infrequently documented in plant surveys [11]. Baskin and Baskin [57] summarized the impact of inbreeding depression on seed germination across 743 instances involving 233 species from 64 families. They found that in 50.1% of the cases, inbred and outcrossed seeds germinated at comparable frequencies, while 8.1% of inbred ones exhibited better germination rates than outcrossed seeds. Interestingly, the authors observed no strong correlation between decreased germination rates and increased *F*_IS_ nor between increased germination rates and heightened levels of population genetic diversity. However, we observed concordant genetic diversity patterns in food-deceptive, allogamous, and pollinator-dependent populations, although these were also self-compatible with the mixed mating systems of *D. majalis*, *D. incarnata* var. *incarnata*, and *D. fuchsii.* Genetic diversity within studied *Dactylorhiza* populations was shaped at a relatively high level comparable to the data reported by Naczk et al. [49] and Hedrén and Nordström [44,58], suggesting that studied *Dactylorhiza* populations can be found via genetically different individuals and/or gene flow via leptokurtic dispersal. The genetic differentiation among them was low and significant (0.080–0.163), showing that gene flow (historical) in northeastern Poland was relatively high or populations were established from one source. However, the isolation processes of these *Dactylorhiza* populations were observed, resulting in the formation of a substructure.

Furthermore, the inbred population was shaped from moderate to high levels in three *Dactylorhiza* taxa, similarly to the studies of Hedrén and Nordström [44], Filippov et al. [59], and Naczk et al. [49]. Meanwhile, in *D. majalis* as the allotetraploid, *F*_IS_ exhibited a wide range of values in the populations reported by Balao et al. [60], Hedrén and Nordström [44], and Naczk and Ziętara [61]. Let us assume the prediction that inbreeding is solely the result of mating among neighbouring plants. In this case, we expect *F*_IS_ to be approximately equal to *F*_ij_ at the smallest distance interval in the studied *Dactylorhiza* populations [6]. In our survey, *F*_IS_ was substantially higher than *F*_ij_ with respect to the first class of spatial distances in most *Dactylorhiza* populations, suggesting that selfing is at least partially responsible for homozygosity [56,62]. However, spontaneous autogamy in three *Dactylorhiza* taxa existed until 1% of pollination in the studied populations [47,48]. Hence, the only explanation of selfing in three *Dactylorhiza* taxa is the pollinator behaviour of bumblebees and other pollinators, which are known to promote geitonogamy and/or autogamy, explaining the development of selfed seeds [40,41]. The important factor shaping *F*_IS_ was the slightly higher selfing frequency compared to outcross seeds germinated in in vitro treatments in *D. majalis* and *D. incarnata* var. *incarnata*, while in *D. fuchsii*, the germination pattern was reversed [42]. This phenomenon suggested that inbred and outbred *D. majalis* and *D. incarnata* var. *incarnata* seeds germinated at a similar or even slightly higher frequency. We stress the careful interpretation of the relationship between seed germination and *F*_IS_. This needs to be confirmed in further studies, including the growth and mortality observations of plants germinated from selfed and outcrossed seeds in the following stages. However, our data are related to a single studied *Dactylorhiza* species, and we can suppose that in *D. fuchsii*, a similar pattern exists in two out of three populations, such as *D. majalis* and *D. incarnata* var. *incarnata*. In the CM and BR populations, high inbreeding and slightly lower kinship coefficients supported the possibility of selfing (geitonogamy). The interesting question is whether biparental inbreeding can exist in food-deceptive *Dactylorhiza* taxa, even though pollinators spend a short time period on flowers and inflorescence and learn to avoid deceptive flowers. They typically only visit fewer flowers per plant and/or a few flowers between inflorescences within populations, promoting cross-pollination and skipping more plants between plant visits. In light of this outcrossing hypothesis [15], biparental inbreeding is rather unlikely. Using videotaping, Ostrowiecka et al. [40] observed that *Apis mellifera* visited three to five flowers on the same inflorescence within 11 to 40 s, contributing to geitonogamy. Our observations noted that *A. mellifera* pollinators did not return to the same flowers and avoided visiting all flowers on the inflorescences. Conversely, the bending of pollinaria serves as a mechanism to prevent geitonogamy and biparental inbreeding among closely related individuals. In *Dactylorhiza*, the average bending time is 39–54 s, which is considered relatively long for deceptive plants and similar to other deceptive *Dactylorhiza* taxa [63]. The bending time observed in each studied *D. majalis* population ranged from 8 s to 2 min and 5 s [40]. This relatively short bending time may be an opportunity for geitonogamy. This observation and the bending times in the studied *Dactylorhiza* populations support our hypothesis that geitonogamy cannot be completely ruled out in deceptive orchids compared to biparental inbreeding. *Dactylorhiza* seems to possess a more generalized pollination system, and numerous pollinators have been described and studied in detail. These pollinators can spend different amounts of time on the flowers, promoting geitonogamy.

Hand pollination using emasculated flowers was employed to assess the extent of apparent geitonogamy occurring via pollinators [64,65]. A previous fruit set observation from controlled pollination in three *Dactylorhiza* documented a moderate level of fruit set (35.4–40.5%). Simultaneously, emasculation experiments in their populations showed a significant decrease in fruiting between these treatments (*D. majalis*, 28.2% fruit set from emasculated flower, paired t = 2.68, df = 8, *p* < 0.002; *D. incarnata* var. *incarnata*, 14.6% fruit set from emasculated flower, paired t = 3.46, df = 10, *p* < 0.006; *D. fuchsii*, 28.2% fruit set from emasculated flower, paired t = 4.83, df = 10, *p* < 0.0007; Wróblewska et al. unpublished data [66]). This study concludes that selfing in three *Dactylorhiza* occurs mainly through geitonogamy. Kropf and Renner [17] have also pointed out the high levels of geitonogamous pollination in *Dactylorhiza*; measuring biparental inbreeding can be challenging in deceptive plants.

The *F*_IS_ observed at the adult stage enabled the assessment of inbreeding depression impacts on the population’s genetic structure. In long-lived plants, *F*_IS_ reflects inbreeding not only in the current generation but also in previous overlapping generations. However, other factors, such as the long lifespan of plants, can affect inbreeding depression [11]. In *D. majalis*, selfing (e.g., geitonogamy) and/or progeny and likely seed dispersal in the vicinity of the mother plant can manifest most strongly in spatial genetic structures. In the case of *D. majalis*, the results of the present study are inconsistent with those of Husband and Schemske [67], who concluded that purging is a significant evolutionary force in natural populations. Without reducing the genetic load, such fixation could reduce inbreeding depression [67,68]. However, inbreeding depression may be lower in long-standing populations with inbreeding than in populations with outcrossing, where selection may have purged the genome of its genetic load [67,68,69,70]. These two alternative approaches in a laboratory should be tested at a later stage of the life cycle of *D. majalis*, such as in seedlings and adult reproductive individuals.

## 5. Conclusions

Selfing (meaning of geitonogamy) and a strong fine-scale genetic structure may have additional and unexplored evolutionary consequences in *Dactylorhiza*, and combined with low inbreeding depression, they may strongly influence the establishment of inbred lines in the cases of *D. majalis* and *D. incarnata* var. *incarnata*. Currently, we cannot state that inbreeding depression may be widely viewed as the primary selective factor allowing transitions to complete selfing in *Dactylorhiza*. On the other hand, there are still limited studies on breeding systems and pollinator behaviour in deceptive multi-flowered orchids, which could shed light on geitonogamy and biparental inbreeding. Our study stressed that different *Dactylorhiza* food-deceptive taxa with varying levels of inbreeding depression can characterize similar FSGSs and inbreeding coefficients. Despite these distinct patterns of inbreeding depression, FSGSs comprise the formation of local family structures in *Dactylorhiza* taxa due to limited gene dispersal (e.g., seeds) and geitonogamy.

## Figures and Tables

**Table 1:** Locations of *D. majalis* (*DM*), *D. incarnata* var. *incarnata* (*DI*), and *D. fuchsii* (*DF*) populations in NE Poland and summary statistics of the genetic diversity and spatial genetic structure estimated using SPAGeDi 1.4 [6]. *N*—number of AFLP samples; *PL*%—frequency of polymorphic loci; *H*—Nei’s gene diversity; *F*_IS_—inbreeding coefficient; CI—the upper and lower 99% confidence interval values; *F_ij_*_(1)_—mean pairwise kinship coefficient among individuals at the first distance class; *b*_1_—regression slope of pairwise kinship at the first distance; *S*_p_—the intensity of the FSGS according Veckemans and Hardy [6]. Voucher specimens were collected by Ada Wróblewska and deposited in the herbarium of the Faculty of Biology, University of Bialystok, Poland. * *p* < 0.05.

Taxa	Population	*GPS*	*N*	*PL*%	*H*	*F*_IS_ (CI)	*F_ij_*_(1)_	*b*_1_	*S*_p_
*DM*	KA	52°53′00″ N 23°40′29″ E	49	62.2	0.205	0.293 (0.000–1.000)	0.095 *	−0.051 *	0.056
	SKI	52°49′50″ N 23°43′10″ E	59	59.6	0.205	0.312 (0.000–1.000)	0.038 *	−0.009 *	0.001
	SKII	52°49′50″ N 23°43′10″ E	54	40.4	0.140	0.192 (0.000–1.000)	0.071 *	−0.021 *	0.022
*DI*	ZB	53°29′02″ N 22°59′28″ E	48	58.6	0.217	0.179 (0.101–0.284)	0.008	−0.002	0.0002
	RO	53°54′39″ N 22°56′32″ E	48	58.6	0.197	0.071 (0.022–0.149)	0.224 *	−0.055 *	0.063
	MR	53°47′25″ N 22°57′22″ E	33	58.1	0.206	0.098 (0.032–0.218)	0.092 *	−0.037 *	0.041
*DF*	CM	52°41′03″ N 23°39′07″ E	58	68.4	0.234	0.113 (0.034–0.244)	0.078 *	−0.021 *	0.019
	BR	52°50′59″ N 23°53′40″ E	57	63.9	0.211	0.134 (0.068–0.226)	0.084 *	−0.026 *	0.028
	GR	53°60′68″ E 22°84′68″ N	49	56.7	0.197	0.079 (0.024–0.169)	−0.008	0.002	−0.0002

## Data Availability

All data cited in the study are publicly available.
